# Antiparasitic Activity of Natural and Semi-Synthetic Tirucallane Triterpenoids from *Schinus terebinthifolius* (Anacardiaceae): Structure/Activity Relationships

**DOI:** 10.3390/molecules19055761

**Published:** 2014-05-05

**Authors:** Thiago R. Morais, Thais A. da Costa-Silva, Andre G. Tempone, Samanta Etel T. Borborema, Marcus T. Scotti, Raquel Maria F. de Sousa, Ana Carolina C. Araujo, Alberto de Oliveira, Sérgio Antônio L. de Morais, Patricia Sartorelli, João Henrique G. Lago

**Affiliations:** 1 Instituto de Ciências Ambientais, Químicas e Farmacêuticas, Universidade Federal de São Paulo, Diadema, SP 09972270, Brazil; 2 Centro de Parasitologia, Instituto Adolfo Lutz, São Paulo, SP 01246902, Brazil; 3 Centro de Ciências Aplicadas e Educação, Universidade Federal da Paraíba, Rio Tinto, PB 58297000, Brazil; 4 Instituto de Química, Universidade Federal de Uberlândia, Uberlândia, MG 38400902, Brazil

**Keywords:** *Schinus terebinthifolius*, leaves, tirucallane triterpenoids, *Leishmania (L.) infantum*, *Trypanosoma cruzi*, structure-activity

## Abstract

Leishmaniasis and Chagas are diseases caused by parasitic protozoans that affect the poorest population in the World, causing a high mortality and morbidity. As a result of highly toxic and long-term treatments, the discovery of novel, safe and more efficacious drugs is essential. In this work, the *in vitro* antiparasitic activity and mammalian cytotoxicity of three natural tirucallane triterpenoids, isolated from leaves of *Schinus terebinthifolius* (Anacardiaceae), and nine semi-synthetic derivatives were investigated against *Leishmania (L.) infantum* and *Trypanosoma cruzi*. Trypomastigotes of *T. cruzi* were the most susceptible parasites and seven compounds demonstrated a trypanocidal activity with IC_50_ values in the range between 15 and 58 µg/mL. Four compounds demonstrated selectivity towards the intracellular amastigotes of *Leishmania*, with IC_50_ values in the range between 28 and 97 µg/mL. The complete characterization of triterpenoids was afforded after thorough analysis of nuclear magnetic resonance (NMR) data as well as electrospray ionization mass spectrometry (ESI-MS). Additionally, structure-activity relationships were performed using Decision Trees.

## 1. Introduction

Specimens of *Schinus terebinthifolius*, known in Brazil as “aroeira-vermelha” or “aroeira-pimenteira”, are large trees that can reach 40 m height and 1–3 m in diameter [1,2]. In folk medicine, this plant has been used to treat ulcers, respiratory problems, wounds, rheumatism, gout, diarrhea, skin disease and arthritis, as well as antiseptic and anti-inflammatory [3]. In addition, decoctions of flowers, stems, leaves and fruits are used for the treatment of tumors and hanseniasis [4].

Previous chemical studies with leaves extracts of *S. terebinthifolius* have been carried out and fatty acids and terpenoids were isolated, especially tirucallane derivatives (masticadienoic acid and schinol) which have shown inhibitory activity on phospholipase A2 [5] and antifungal potential against *Paracoccidioides brasiliensis* [6]. Other compounds, such as phenolic derivatives (gallic acid, methyl and ethyl gallates) and flavonoids (*trans*-catechin, kaempferol, quercitrin, afzelin, myricetin, and myricetrin) were also isolated from the leaves and displayed antiradical and cytotoxic activities [7,8]. Chemical analysis of barks of *S. terebinthifolius* indicated the presence of anthraquinones, xanthones and steroids [9]. Additionally, essential oils from leaves, flowers and fruits of *S. terebinthifolius* were also analyzed, and proved to be composed of mono- and sesquiterpenes. With regards to the evaluation of the biological activity, the volatile oils of fruits showed allelopathic, cytotoxic and trypanocidal activities, while the leaf oil showed cytotoxic activity [10,11,12,13,14,15]. Furthermore, previous work demonstrated anti-*Leishmania amazonensis* (promastigotes) activity of the aqueous and hydro-alcoholic extracts from leaves of *S. terebinthifolius* [16]. Considering other plants from the genus *Schinus*, the MeOH extract of the leaves and fruits of *S. molle* have also shown activity against *L. infantum*, *Trypanosoma brucei*, *T. cruzi*, and *Plasmodium falciparum* [17].

Leishmaniasis and Chagas’ disease are parasitic diseases caused by the protozoans *Leishmania spp.* and *Trypanosoma cruzi*, respectively*.* They are recognized by World Health Organization among the World’s most neglected diseases, affecting millions of people [18]. Considering the limited and highly toxic therapeutic arsenal, the study of alternative therapies is essential. In continuation of the investigation of bioactive compounds from Brazilian flora [19,20,21,22], the present study was undertaken to determine the effectiveness and cytotoxicity of three main compounds isolated from leaves extract of *S. terebinthifolius* (*E*- and *Z*-masticadienoic acids and *Z*-schinol) against promastigotes and intracellular amastigotes of *Leishmania (L.) infantum,* as well as trypomastigote forms of *Trypanosoma cruzi.* Aiming to establish relationships between the chemical structures and the antiparasitic activity, nine semi-synthetic tirucallane derivatives were obtained and tested against parasites and mammalian cells.

## 2. Results and Discussion

**Figure 1:**
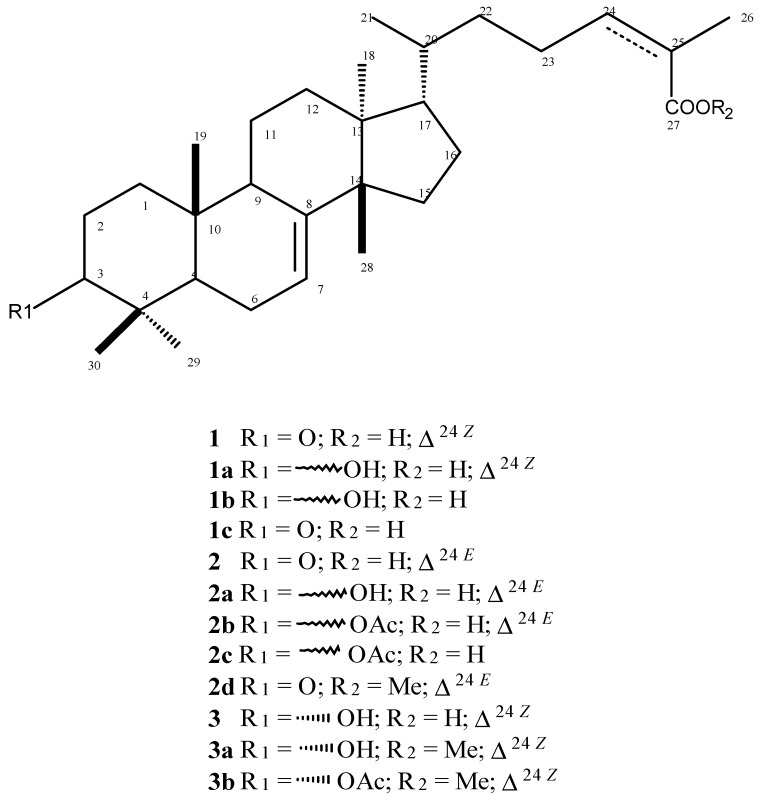
Structures of natural compounds **1**–**3** and semi-synthetic **1a**–**c**, **2a**–**d**, **3a**, and **3b** tirucallane triterpenoids.

*Schinus terebinthifolius* is a Brazilian plant that produces great amounts of tirucallane derivatives such as (*Z*)-masticadienoic (**1**) and (*E*)-masticadienoic (**2**) acids as well as (*Z*)-schinol (**3**). In this work, these compounds were isolated from leaves of this plant using several chromatographic techniques and their structures were confirmed by NMR and ESI-MS spectral analysis and comparison with data described in the literature [5,23]. The triterpenoids **1**–**3** were subjected to different reactions: reduction of carbonyl group at C-3, methylation of carboxyl group at C-27, acetylation of hydroxyl group at C-3 and hydrogenation of double bond at C-24, to afford nine derivatives (**1a**–**c**, **2a**–**d**, **3a**, and **3b**–Figure 1), being **1c**, **2c**, **2d**, **3a**, and **3b** new compounds. The structures of these compounds were confirmed by MS and ^13^C-NMR by the comparison of respective spectral data with those recorded for **1**–**3**. In the case of carbonyl reduction (compounds **1a** and **2a**) the absence of a peak at δ 217 associated to the occurrence of a signal at range δ 79–76 indicates the presence of an oxymethine carbon at C-3. The ^13^C-NMR spectra of derivatives **2b**, **2c** and **3b** showed additional peaks assigned to carbonyl (δ 170) and methyl (δ 20) carbons of acetyl group at C-3. The hydrogenated derivatives **1b**, **1c** and **2c** showed two additional units in the ESI-MS mass spectra in comparison with **1** and **2b**. This data, associated to the absence of peaks assigned to C-24 and C-25 at δ 146 and 126, respectively, indicated that the hydrogenation occurs exclusively at Δ^2^^4^. Finally, structures of methyl esters **2d** and **3a** were confirmed by the presence of additional peaks at δ 52 in the respective ^13^C-NMR spectra.

The antileishmanial and antitrypanosomal activities of three natural and nine semi-synthetic derivatives of tirucallane triterpenoids were evaluated against *L. (L.) infantum* and *T. cruzi*. According to the colorimetric assay of MTT and light microscopy, seven compounds killed 100% of trypomastigote forms of *T. cruzi* at the highest tested concentration, resulting in IC_50_ values in the range of 15.75 to 58.36 µg/mL (Table 1).

**Table 1:** Antiparasitic (antileishmanial and antitrypanosomal) and cytotoxic effects of natural compounds **1**–**3**, semi-synthetic **1a**–**c**, **2a**–**d**, **3a**, and **3b** and standards.

IC_50_ (g/mL) ^a^ CI95%				CC_50_ (μg/mL) ^b^ CI95%	SI	
Compounds	*L. infantum*	*L. infantum*	*T. cruzi*	NCTC	AMA^c^	TRY^d^
	Promastigotes	Amastigotes	Trypomastigotes			
**1**	NA	66.51 (47.68–92.79)	NA	>200	>3	–
**1a**	NA	NA	20.18 (16.70–24.39)	69.50 (64.01–75.45)	–	3.4
**1b**	NA	NA	17.64 (15.97-19.50)	76.39 (70.02-83.33)	–	4.3
**1c**	NA	NA	NA	>200	–	–
**2**	NA	64.90 (41.48–101.50)	15.75 (9.80–25.30)	96.48 (77.38–120.30)	1.5	6.1
**2a**	NA	97.59 (89.82–106.00)	29.59 (25.61–34.18)	95.49 (65.22–139.80)	1.0	3.2
**2b**	NA	NA	58.36 (42.82–79.55)	69.31 (38.74–82.64)	–	1.1
**2c**	NA	NA	49.20 (41.69–58.05)	57.78 (56.10–59.52)	–	1.2
**2d**	NA	NA	NA	>200	–	–
**3**	57.82 (54.01–61.91)	28.95 (19.87–42.16)	16.28 (8.94–29.60)	69.50 (64.01–75.45)	2.4	4.3
**3a**	NA	NA	NA	>200	–	–
**3b**	NA	NA	NA	>200	–	–
**miltefosine**	6.87	7.25	–	49.72	–	–
**benznidazole**	–	–	114.68	–	–	–

IC_50_: 50% inhibitory concentration; CC_50_: 50% cytotoxic concentration (mammalian cells); NA: not active*;* CI95%: 95% Confidence Interval; SI AMA: selectivity index amastigotes (CC_50_ mammalian cells/IC_50_ *Leishmania* amastigotes); SI TRY: selectivity index trypomastigotes (CC_50_ mammalian cells/IC_50_ trypomastigotes).

Despite the lack of activity of compound **1**, the semi-synthetic derivatives **1a** and **1b** demonstrated antitrypanosomal activity, with IC_50_ values of 20.18 and 17.64 µg/mL, respectively. In addition, an enhanced mammalian toxicity was also observed. Otherwise, compound **2**, the most effective against the trypomastigotes (IC_50_ 15.75 µg/mL), demonstrated a higher activity when compared to the semi-synthetic derivatives **2a**, **2b** and **2c**, which showed moderate inhibitory effects and IC_50_ values between 29.59 and 58.36 µg/mL. Considering the 95% confidence intervals, the mammalian toxicity of **2a**, **2b** and **2c** was comparable to that of the natural prototype **2**. Likewise, the natural compound **3**, which showed an IC_50_ value of 16.28 µg/mL against trypomastigotes, also rendered less effective derivatives after methylation at C-27 (**3a**) and acetylation at C-3 (**3b**), although the derivatization resulted in no more cytotoxic compounds when compared to the natural compound **3**. Benznidazole was used as a standard drug and gave an IC_50_ value of 114.68 µg/mL [21]. Considering the relation between the antiparasitic activity and mammalian cytotoxicity, given by the selectivity index (CC_50_/IC_50_), compounds **2**, **3** and the semi-synthetic derivative **1b** demonstrated the highest indexes, ranging from 4 to 6. According to the Food and Drug Administration guidance for the development of drugs [24], it is desirable to have a high selectivity index giving maximum activity with minimal cell toxicity.

Among the twelve tested compounds, only the natural prototype **3** showed effectiveness against *L. infantum* promastigotes, with an IC_50_ value of 57.82 µg/mL. The modifications of the semi-synthetic tirucallane triterpenoid derivatives (compounds **1a**–**1c**, **2a**–**2d**, **3a** and **3b**) showed no improvement of the antileishmanial effectiveness against the extracellular forms of *L. infantum*. However, when tested against the intracellular amastigotes, four compounds (**1**, **2**, **2a** and **3**) resulted in IC_50_ values in the range of 28.95 to 97.59 µg/mL. This effect could be ascribed to a possible macrophage activation, which could also have contributed to an oxygen burst and up-regulation of cytokines by host cells [25]. The possible immunomodulatory effect of these compounds may be investigated in future assays. Similarly, the pentavalent antimonial glucantime, the main clinical drug in use for leishmaniasis has also shown no effectiveness against the extracellular forms of the parasite, and its antiparasitic activity has been attributed to host cell activation [26]. Except for compound **1**, which showed no mammalian toxicity up to the highest tested concentration (>200 µg/mL), compounds **2** and **3** resulted in CC_50_ values of 96 and 69 µg/mL, respectively. Considering the selectivity index, compound **1** was the most promising candidate without toxicity to NCTC cells, showing a value higher than **3**. Miltefosine showed a 50% cytotoxic concentration (CC_50_) of 49.72 µg/mL and resulted in a SI of 7. Miltefosine was used as a standard drug against promastigotes and amastigotes, with IC_50_ values of 6.87 and 7.25 µg/mL, respectively [27].

**Figure 2:**
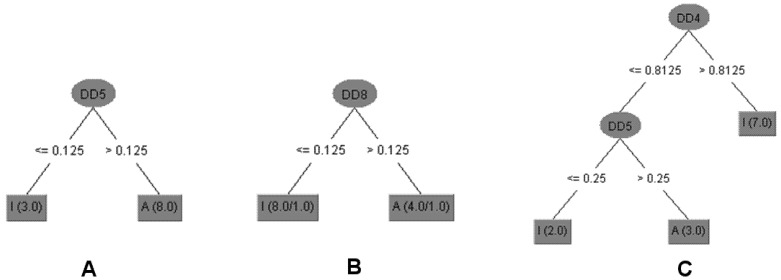
Decision Trees (DT) generated for the set of triterpenoids with antiparasitic activity. (**A**) The compounds with higher values of DD5 (differences of the hydrophobic volumes at energy level of −1.0 kcal/mol) than 0.125 were active against *T. cruzi* trypomastigotes. (**B**) Compounds with higher values of DD8 (differences of the hydrophobic volumes at energy level of −1.6 kcal/mol) than 0.125 were active against *L. infantum* amastigotes. (**C**) Triterpenoids with DD4 (differences of the hydrophobic volumes at energy level of −0.8 kcal/mol) values higher than 0.8125 or DD5 values lower or equal to 0.25 were not cytotoxic against NCTC. The numbers in brackets show the number of compounds correctly classified/incorrectly as active (A) or inactive (I).

The decision tree (DT) model (Figure 2) selected with the aim of establishing relationships between chemical structures and antitrypanosomal activity of compounds **1a**–**c**, **2a**–**d**, **3a** and **3b** used the DD5 descriptor. This molecular descriptor quantifies the variation between the maximum hydrophobic volume obtained upon variation of ligand conformation and the hydrophobic volume of the imported three dimensional structure into Volsurf at a Molecular Interaction Fields (MIF) energy value of −1.0 kcal/mol [28].

Triterpenoids **1**, **1c** and **2d** displayed equal or lower DD5 values than 0.125. Thus, it is possible to infer that compounds with a carbonyl group at C-3 with *Z* configuration at Δ^24^, with an ester group at C-27, or without a double bond between C-24/C-25 showed lower differences of hydrophobic volumes and are inactive against the *T. cruzi*. The DT model predicts for all 12 compounds (100%) of antitrypanosomal activity for the training set and 11 compounds (92.9%) for internal validation (Table 2). The obtained data also suggested that the stereochemistry of hydroxyl group at C-3 to triterpenoids **3** and **1a** was determinant to antileishmanial activity (promastigote and amastigote forms of *L. infantum*) since these compounds displayed the same planar structure but different configuration. However, using a DT model with only one descriptor as DD8 (differences of the hydrophobic volumes at energy level −1.6 kcal/mol) it was possible to predict activity against *L. infantum* amastigotes for 10 compounds (83.3%) of the training set and internal validation (Table 2) [29]. The data showed that compounds with higher values of DD8 than 0.125 were active. Triterpenoids with carbonyl or hydroxyl at C-3, double bond at C-24, and carboxylic acid at C-27, showed higher values of DD8 (compounds **1**, **1a**, **2** and **3**). It is important to highlight that the prediction activity error of compound **1a** was due to DD8, which is a three dimensional descriptor that does not encode directly the stereochemistry but the difference of 3D conformations of the compounds.

**Table 2:** Comparison of experimental antileishmanial, antitrypanosomal and cytotoxic activities with data predicted by Decision tree models for the training set and internal cross validation (leave-one-out).

*T. cruzi* (Trypomastigotes)				*L. infantum* (Amastigotes)			NCTC		
Compounds	Experimental	Train	Validation	Experimental	Train	Validation	Experimental	Train	Validation
**1**	I	I	I	A	A	A	I	I	I
**2**	A	A	A	A	A	A	I	I	I
**2a**	A	A	A	A	I	I	I	I	I
**1a**	A	A	A	I	A	A	A	A	A
**1b**	A	A	A	I	I	I	A	A	I
**2b**	A	A	A	I	I	I	I	I	I
**2d**	I	I	A	I	I	I	I	I	A
**3**	A	A	A	A	A	A	A	A	A
**1c**	I	I	I	I	I	I	I	I	A
**2c**	A	A	A	I	I	I	I	I	I
**3a**	A	A	A	I	I	I	I	I	I
**3b**	A	A	A	I	I	I	I	I	I

Cytotoxicity of triterpenoids was also related to the difference of hydrophobic values (Figure 2) once descriptors DD4 and DD5 were selected and compounds with higher values than 0.8125 to DD4 (differences of the hydrophobic volumes at energy level of −0.8 kcal/mol) or lower or equal values than 0.25 to DD5 were not cytotoxic against NCTC. DT model predicted 100% of cytotoxicity of the training set and 75% of internal validation (Table 2). Therefore, the cytotoxic compounds displayed hydroxyl group at C-3 and *Z* configuration or absence of double bond between C-24 and C-25 with carboxylic acid at C-27 (compounds **1a**, **1b** and **3**).

## 3. Experimental

### 3.1. General Experimental Procedures

All solvents used were of analytical grade and purchased from CAAL (São Paulo, Brazil). Silica gel (230–400 mesh, Merck, Darmstadt, Germany) and Sephadex LH-20 (Aldrich, St. Louis, MO, USA) were used for column chromatographic separation, while silica gel 60 F_254_ (Merck) was used for analytical TLC (0.25 mm). ^1^H and ^13^C spectra were recorded, respectively, at 300 and 75 MHz in a Bruker Ultrashield 300 Advance III spectrometer. CDCl_3_ (Aldrich) was used as solvent and the residual peak of the non-deuterated solvent as internal standard. Chemical shifts (δ) are reported in ppm and coupling constant (*J*) in Hz. ESI-MS were measured with a Platform II mass spectrometer (Micromass, MA, USA), operating in negative mode.

### 3.2. Plant Material

Leaves of *S. terebinthifolius* were randomly collected from an individual tree at Mogi-Guaçu region (São Paulo State, Brazil) on February/2010 by Dr. Maria Claudia Marx Young from *Instituto de Botânica* (São Paulo, Brazil), where a reference specimen (SP272591) was deposited.

### 3.3. Extraction and Isolation of Natural Triterpenoids 1–3

The leaves of *S. terebinthifolius* were dried at 40 °C during 7 days. After grinding, the plant material (1 kg) was extracted with *n*-hexane (10 × 1 L) for removal of fatty material. The remaining material was submitted to an exhaustive extraction with EtOH at room temperature using an accelerated solvent extractor system (Dionex ASE-350). Distillation of the solvent under reduced pressure yielded the crude EtOH extract, which was partitioned between EtOH-H_2_O 1:2 (500 mL) and hexane (4 × 250 mL) to afford 22 g of *n*-hexane phase after solvent removal under reduced pressure. Thus, part of the *n*-hexane phase (20 g) was subjected to fractionation by silica gel column chromatography and eluted with increasing amount of EtOAc in *n*-hexane to afford 37 fractions (125 mL each). After analysis by TLC, these fractions were pooled into eight groups (A-H). Group E (1,001 mg) was subjected to fractionation on a Sephadex LH-20 column (3 × 50 cm–flow 1.0 mL/min) and eluted with MeOH, yielding 30 fractions (3 mL each), which were pooled into six groups after analysis by TLC (E1 to E6). Groups E2 (310 mg) and E3 (500 mg) consisted of **2** and **1**, respectively. Group F (1800 mg) was subjected to separation on a Sephadex LH-20 column (3 × 60 cm–flow 0.7 mL/min) and eluted with MeOH*,* yielding 40 fractions (3 mL each), which, after monitoring by TLC, were pooled into four groups (C1 to C4). Group C2 (1020 mg) was subjected to fractionation on a Sephadex LH-20 column (3 × 50 cm–flow 1.0 mL/min) eluted with MeOH, yielding 27 fractions (3 mL each), which were pooled into four groups (C2/1 to C-2/4) after analysis by TLC. Purification of group C2/3 (580 mg) by silica gel column chromatography (3 × 40 cm), using increasing amounts of EtOAc in hexane, allowed the isolation of **3** (90 mg).

*3-Oxotirucalla-7,24Z-dien-27-oic acid* (*Z-masticadienoic acid*, **1**). Amorphous solid. ESI-MS *m/z* 453 [M−H]^−^; ^1^H-NMR (CDCl_3_), δ/ppm: 6.02 (t, *J* = 6.0 Hz, H-24), 5.30 (t, *J* = 3.0 Hz, H-7), 2.77 (td, *J* = 15.1 and 6.0 Hz, H-3), 2.56 (m, H-23), 2.27 (m, H-5), 2.23 (m, H-2), 2.09 (m, H-6), 1.97 (m, H-16), 1.90 (s, CH_3_-26), 1.89 (m, H-9), 1.57 (m, H-22), 1.54 (m, H-20), 1.52 (m, H-15), 1.50 (m, H-12), 1.47 (m, H-17), 1.41 (m, H-1), 1.12 (s, CH_3_-28), 1.05 (s, CH_3_-30), 1.00 (s, CH_3_-29), 0.89 (m, H-11), 0.88 (d, *J* = 6.0 Hz, CH_3_-21), 0.87 (s, CH_3_-18), 0.77 (s, CH_3_-19). ^13^C-NMR (CDCl_3_), δ/ppm: 217.1 (C-3), 173.4 (C-27), 146.2 (C-24), 146.1 (C-8), 126.5 (C-25), 117.8 (C-7), 52.9 (C-17), 51.2 (C-14), 48.6 (C-5), 48.4 (C-9), 47.9 (C-4), 43.5 (C-13), 38.5 (C-1), 36.1 (C-20), 35.7 (C-10 and C-15), 35.0 (C-2), 34.1 (C-12), 33.8 (C-22), 28.2 (C-16), 27.4 (C-28), 27.3 (C-29), 26.9 (C-23), 25.4 (C-30), 24.5 (C-6), 20.6 (C-26), 18.3 (C-18), 18.2 (C-11), 18.0 (C-21), 13.0 (C-19).

*3-Oxotirucalla-7,24E-dien-27-oic acid (E-masticadienoic acid*, **2**)*.* Amorphous solid. ESI-MS *m/z* 453 [M−H]^−^; ^1^H-NMR (CDCl_3_), δ/ppm: 6.06 (t, *J* = 6.9 Hz, H-24), 5.30 (m, H-7), 2.77 (td, *J* = 15.0 and 6.0 Hz, H-2), 2.56 (m, H-23), 2.24 (m, H-2), 2.10 (m, H-6), 2.09 (m, H-5), 2.03 (m, H-16), 2.00 (m, H-9), 1.92 (s, CH_3_-26), 1.73 (m, H-15), 1.60 (m, H-12), 1.57 (m, H-11), 1.49 (m, H-1), 1.47 (m, H-20), 1.42 (m, H-17), 1.30 (m, H-22), 1.12 (s, CH_3_-19), 1.04 (s, CH_3_-29), 1.00 (s, CH_3_-28 and CH_3_-30), 0.89 (d, *J* = 6.0 Hz, CH_3_-21), 0.81 (s, CH_3_-18). ^13^C-NMR (CDCl_3_), δ/ppm: 217.1 (C-3), 173.1 (C-27), 145.6 (C-24), 145.8 (C-8), 126.7 (C-25), 117.9 (C-7), 52.2 (C-17), 51.1 (C-14), 52.8 (C-5), 47.8 (C-4), 43.5 (C-13), 48.5 (C-9), 38.5 (C-1), 36.0 (C-20), 35.0 (C-23), 34.9 (C-10), 34.6 (C-2), 34.0 (C-15), 33.6 (C-12), 26.0 (C-22), 28.2 (C-16), 27.4 (C-30), 24.5 (C-28), 24.3 (C-6), 21.9 (C-18), 21.6 (C-29), 18.2 (C-11), 18.1 (C-21), 12.7 (C-19), 11.9 (C-26).

*3α-Hydroxytirucalla-7,24Z-dien-27-oic acid* (*Z-schinol*, **3**). Amorphous solid. ESI-MS *m/z* 455 [M−H]^−^; ^1^H-NMR (CDCl_3_), δ/ppm: 6.07 (t, *J* = 6.3 Hz, H-24), 5.28 (m, H-7), 3.46 (dd, *J* = 10.2 and 5.4 Hz, H-3), 2.50 (m, H-9), 2.46 (m, H-23), 2.05 (s, CH_3_-26), 2.04 (m, H-2), 1.97 (m, H-16), 1.95 (s, CH_3_-30), 1.91 (m, H-5), 1.90 (m, H-6), 1.59 (m, H-12), 1.56 (m, H-11), 1.46 (m, H-1), 1.43 (m, H-15), 1.42 (m, H-17), 1.39 (m, H-20), 1.37 (m, H-22), 0.93 (s, CH_3_-28), 0.90 (s, CH_3_-29), 0.89 (d, *J* = 6.0 Hz, CH_3_-21), 0.83 (s, CH_3_-18), 0.77 (s, CH_3_-19). ^13^C-NMR (CDCl_3_), δ/ppm: 173.6 (C-27), 146.1 (C-24), 146.0 (C-8), 125.7 (C-25), 117.8 (C-7), 76.4 (C-3), 53.3 (C-17), 51.6 (C-14), 48.6 (C-9), 44.5 (C-5), 43.9 (C-13), 37.4 (C-4), 36.4 (C-2), 36.2 (C-1 and C-20), 36.1 (C-22), 35.6 (C-10), 34.7 (C-12), 34.0 (C-15), 28.2 (C-16 and C-28), 27.4 (C-30), 26.9 (C-23), 24.3 (C-6), 22.5 (C-29), 21.4 (C-18), 20.6 (C-26), 18.7 (C-21), 18.5 (C-11), 13.0 (C-19).

### 3.4. Preparation of Semi-Synthetic Compounds

#### 3.4.1. Reduction of the Carbonyl Group at C-3 (Compounds **1** and **2**)

To a solution of **1** (100 mg) or **2** (50 mg) dissolved in MeOH (10 mL) was added NaBH_4_ (42 mg) in small portions and carefully. The reaction mixture was stirred overnight at room temperature. After addition of H_2_O, the solvent was partially evaporated under reduced pressure. The residue was extracted with EtOAc (3 × 25 mL) and the combined organic layers were dried over Na_2_SO_4_, filtered and concentrated. Purification by silica gel chromatography (hexane/EtOAc 7:3) afforded **1a** (41 mg) or **2a** (46 mg).

#### 3.4.2. Hydrogenation of Δ^24^ (Compounds **1**, **1a**, **2b**)

In a high-pressure reactor (stainless steel), was added **1** (20 mg) or **1a** (20 mg) or **2b** (15 mg) and Ni-Raney catalyst (50 mg). After addition of H_2_ (20 atm), the mixture was stirred for 3 h at 100 °C. Then, the product was dissolved in CH_2_Cl_2_ and the catalyst removed by filtration over a bed of Celite. Purification by silica gel chromatography (hexane/EtOAc 9:1) afforded **1c** (15 mg) or **1b** (14 mg) or **2c** (9 mg).

#### 3.4.3. Acetylation of Hydroxyl Group at C-3 (Compounds **2a** and **3a**)

A solution of **2a** (50 mg) or **3a** (15 mg) was dissolved in pyridine (4 mL) and cooled to 0 °C. Acetic anhydride (2 mL) was added and was stirred overnight at room temperature. Excess of reagents were removed under reduced pressure. After addition of H_2_O (5 mL), the residue was extracted with CH_2_Cl_2_ (3 × 25 mL). The organic layer was dried over Na_2_SO_4_, filtered and concentrated. Purification by silica gel chromatography with hexane/EtOAc (7:3) afforded **2b** (35 mg) or hexane/EtOAc (95:5) afforded **3b** (10 mg).

#### 3.4.4. Methylation of Carboxylic Acid (Compounds **2** and **3**)

To a solution of KOH (1.7 g) in H_2_O (2.3 mL) and EtOH (8.3 mL) was added a solution of Diazald (7.2 g) dissolved in Et_2_O (80 mL). This mixture was heated and the product distillated to afford ether solution of diazomethane (0.75 g). Immediately, an excess of diazomethane was added to **2** (70 mg) or **3** (25 mg). The organic layer was dried over Na_2_SO_4_, filtered and concentrated under reduced pressure. Purification by silica gel chromatography (hexane/EtOAc 9:1) afforded compounds **2d** (50 mg) or **3a** (21 mg).

*3ξ-Hydroxytirucalla-7,24Z-dien-27-oic acid* (**1a**). Amorphous solid. ESI-MS *m/z* 455 [M−H]^−^; ^13^C-NMR (CDCl_3_), δ/ppm: 169.2 (C-27), 146.5 (C-24), 144.0 (C-8), 127.3 (C-25), 118.5 (C-7), 79.3 (C-3), 53.3 (C-17), 51.6 (C-14), 49.1 (C-9), 44.8 (C-5), 43.9 (C-13), 37.7 (C-4), 36.5 (C-20), 36.1 (C-22), 35.1 (C-10), 34.4 (C-15), 34.3 (C-12), 31.7 (C-1), 28.6 (C-16), 28.2 (C-28), 27.5 (C-30), 26.9 (C-2), 26.1 (C-23), 24.3 (C-6), 22.0 (C-29 and C-18), 20.9 (C-26), 18.4 (C-21 and C-11), 13.2 (C-19).

*3ξ-Hydroxytirucall-7-en-27-oic acid* (**1b**). Amorphous solid. ESI-MS *m/z* 457 [M−H]^−^; ^13^C-NMR (CDCl_3_), δ/ppm: 181.5 (C-27), 146.1 (C-8), 117.8 (C-7), 76.3 (C-3), 53.0 (C-17), 51.2 (C-14), 48.6 (C-9), 44.5 (C-5), 43.4 (C-13), 39.2 (C-25), 37.4 (C-4), 36.0 (C-20), 35.8 (C-24), 35.0 (C-10), 34.9 (C-22), 34.7 (C-12), 34.0 (C-15), 31.2 (C-1), 28.2 (C-16), 27.7 (C-23 and C-28), 25.3 (C-2), 24.0 (C-6), 21.9 (C-18), 21.8 (C-29), 21.6 (C-30), 18.3 (C-11), 17.9 (C-21), 17.0 (C-26), 12.9 (C-19).

*3-Oxotirucall-7-en-27-oic acid* (**1c**). Amorphous solid. ESI-MS *m/z* 455 [M−H]^−^; ^13^C-NMR (CDCl_3_), δ/ppm: 217.0 (C-3), 182.4 (C-27), 146.0 (C-8), 117.8 (C-7), 53.0 (C-5), 52.3 (C-17), 51.2 (C-14), 48.5 (C-9), 47.9 (C-4), 43.5 (C-13), 38.5 (C-1), 35.8 (C-20 and C-24), 35.7 (C-25), 35.0 (C-10 and C-22), 34.9 (C-2), 34.0 (C-15), 33.7 (C-12), 28.2 (C-16), 27.4 (C-23), 24.5 (C-6 and C-28), 24.1 (C-30), 22.0 (C-18), 21.6 (C-29), 18.3 (C-21 and C-11), 17.0 (C-26), 12.8 (C-19).

*3ξ-Hydroxytirucalla-7,24E-dien-27-oic acid* (**2a**). Amorphous solid. ESI-MS *m/z* 455 [M−H]^−^; ^13^C-NMR (CDCl_3_), δ/ppm: 173.1 (C-27), 146.0 (C-8), 145.6 (C-24), 126.7 (C-25), 117.8 (C-7), 76.4 (C-3), 52.2 (C-17), 51.6 (C-14), 48.6 (C-9), 44.5 (C-5), 43.9 (C-13), 37.4 (C-4), 36.4 (C-2), 36.1 (C-1), 36.0 (C-20), 35.6 (C-10), 35.0 (C-23), 34.7 (C-12), 34.0 (C-15), 28.2 (C-16), 27.4 (C-30), 26.0 (C-22), 24.5 (C-28), 24.3 (C-6), 21.9 (C-18), 21.6 (C-29), 18.5 (C-11), 18.1 (C-21), 12.7 (C-19), 11.9 (C-26).

*3ξ-Acetoxytirucalla-7,24E-dien-27-oic acid* (**2b**). Amorphous solid. ESI-MS *m/z* 497 [M−H]^−^; ^13^C-NMR (CDCl_3_), δ/ppm: 171.1 (C-27), 171.0 (C=O), 146.0 (C-24), 145.7 (C-8), 126.6 (C-25), 117.7 (C-7), 81.1 (C-3), 52.8 (C-17), 51.2 (C-14), 50.7 (C-5 and C-9), 43.5 (C-13), 37.8 (C-4), 36.8 (C-20), 36.0 (C-1 and C-22), 34.8 (C-10), 34.6 (C-15), 33.9 (C-12), 28.2 (C-16), 27.2 (C-28), 27.0 (C-23), 24.2 (C-2), 23.7 (C-6), 21.8 (C-18 and C-30), 21.4 (C-29), 21.0 (Me), 18.2 (C-21 and C-11), 13.1 (C-19), 11.9 (C-26).

*3ξ-Acetoxytirucall-7-en-27-oic acid* (**2c**). Amorphous solid. ESI-MS *m/z* 499 [M−H]^−^; ^13^C-NMR (CDCl_3_), δ/ppm: 171.1 (C-27), 170.9 (C=O), 145.9 (C-8), 117.6 (C-7), 81.2 (C-3), 53.0 (C-17), 51.1 (C-14), 50.8 (C-5 and C-9), 43.5 (C-13), 37.8 (C-4 and C-25), 36.0 (C-1 and C-20), 35.9 (C-24), 34.8 (C-10 and C-22), 34.7 (C-15), 34.0 (C-12), 28.2 (C-16), 27.4 (C-28), 27.3 (C-23), 24.2 (C-2), 23.8 (C-6), 21.9 (C-18), 21.4 (C-29), 21.0 (Me), 18.3 (C-11), 18.1 (C-21 and C-30), 17.0 (C-26), 13.1 (C-19).

*Methyl 3-oxotirucalla-7,24E-dien-27-oate* (**2d**). Amorphous solid. ESI-MS *m/z* 467 [M−H]^−^; ^13^C-NMR (CDCl_3_), δ/ppm: 216.9 (C-3), 168.6 (C-27), 145.9 (C-8), 144.1 (C-24), 126.5 (C-25), 117.8 (C-7), 52.9 (C-5), 52.8 (OMe), 52.3 (C-17), 51.2 (C-14), 48.5 (C-9), 47.9 (C-4), 43.5 (C-13), 38.5 (C-1), 36.0 (C-20), 35.7 (C-23), 35.0 (C-10), 34.9 (C-2), 34.1 (C-15), 33.6 (C-12), 28.2 (C-16), 26.7 (C-22), 27.4 (C-30), 24.5 (C-28), 24.4 (C-6), 22.0 (C-18), 21.6 (C-29), 18.3 (C-21), 18.2 (C-11), 12.8 (C-19 and C-26).

*Methyl 3α-hydroxytirucalla-7,24Z-dien-27-oate* (**3a**). Amorphous solid. ESI-MS *m/z* 469 [M−H]^−^; ^13^C-NMR (CDCl_3_), δ/ppm: 168.6 (C-27), 145.8 (C-8), 144.1 (C-24), 126.4 (C-25), 117.8 (C-7), 79.3 (C-3), 52.9 (C-5), 52.2 (OMe), 51.1 (C-14), 50.6 (C-17), 48.9 (C-9), 43.6 (C-13), 37.2 (C-4), 36.1 (C-1), 35.7 (C-10 and C-20), 35.6 (C-23), 34.9 (C-2 and C-12), 34.0 (C-15), 28.2 (C-16), 27.7 (C-28), 27.3 (C-30), 26.7 (C-22), 23.9 (C-6), 21.9 (C-18 and C-29), 20.7 (C-26), 18.2 (C-11), 18.1 (C-21), 13.1 (C-19).

*Methyl 3α-acetoxytirucalla-7,24Z-dien-27-oate* (**3b**). Amorphous solid. ESI-MS *m/z* 513 [M−H]^−^; ^13^C-NMR (CDCl_3_), δ/ppm: 171.0 (C-27), 168.6 (C=O), 145.9 (C-8), 144.1 (C-24), 126.4 (C-25), 117.6 (C-7), 81.1 (C-3), 52.9 (C-5), 52.8 (OMe), 51.2 (C-17 and C-14), 48.8 (C-9), 47.6 (C-4), 43.5 (C-13), 38.1 (C-1), 36.0 (C-20), 35.7 (C-23), 34.8 (C-10 and C-2), 34.0 (C-15), 33.7 (C-12), 28.2 (C-16), 27.6 (C-30), 26.7 (C-22), 24.2 (C-6 and C-28), 21.9 (C-18), 21.3 (C-29), 20.7 (C-26), 20.1 (Me), 18.2 (C-11), 18.1 (C-21), 13.1 (C-19).

### 3.5. Bioassay Procedures

BALB/c mice and Golden hamsters (*Mesocricetus auratus*) were supplied by the animal breeding facility at the *Instituto Adolfo Lutz*, São Paulo, Brazil and maintained in sterilized cages under a controlled environment, receiving water and food *ad libitum*. Animal procedures were performed with the approval of the Research Ethics Commission, in agreement with the Guide for the Care and Use of Laboratory Animals from the National Academy of Sciences.

### 3.6. Parasite Maintenance

Isolated promastigotes of *L. (L.) infantum* (MHOM/BR/1972/LD) were maintained in M-199 medium supplemented with 10% fetal bovine serum (FBS) and 0.25% hemin at 24 °C. *L. infantum* was maintained in golden hamsters up to 60–70 days post-infection. Amastigotes were harvested from spleens of infected hamsters by differential centrifugation [30]. *T. cruzi* trypomastigotes (Y strain) were maintained in LLC-MK2 (ATCC CCL 7) cells using RPMI-1640 medium supplemented with 2% FBS at 37 °C 5% CO_2_-humidified incubator [21].

### 3.7. Mammalian Cells

Peritoneal macrophages were collected from the peritoneal cavity of female BALB/c mice by washing with RPMI-1640 without phenol red, supplemented with 10% FBS. NCTC (clone 929) cells were maintained in RPMI-1640 (without phenol red and supplemented with 10% FBS) at 37 °C in a humidified atmosphere containing 5% CO_2_ [31].

### 3.8. Determination of the Activity Against L. infantum-Promastigotes.

To determine the 50% inhibitory concentration (IC_50_) against *L. infantum* promastigotes, compounds **1**–**3**, **1a**–**c**, **2a**–**d**, **3a**, and **3b** were previously dissolved in dimethyl sulphoxide (DMSO) and diluted with M-199 medium in 96-well microplates. Promastigotes were counted in a Neubauer hemocytometer and seeded at 1 × 10^6^/well with a final volume of 150 μL. The tested compounds were incubated in a range concentration of 100 to 0.78 μg/mL for 48 h at 24 °C. The viability of promastigotes was verified by motility and morphology using light microscopy and by the colorimetric MTT assay [32]. Briefly, 3-[4,5-dimethylthiazol-2-yl]-2,5-diphenyltetrazolium bromide (MTT, 5 mg/mL) was dissolved in phosphate-buffered saline (PBS), sterilized through 0.22 µm membranes and added, 20 µL/well, for 4 h at 24 °C. Formazan extraction was performed using 10% sodium dodecyl sulfate (SDS) for 18 h (80 µL/well) at 24 °C and the optical density (OD) was determined in a spectrophotometric plate reader Filter Max 5 (Molecular Devices, Sunnyvale, CA, USA) at 570 nm. Promastigotes incubated without compounds or DMSO were used as control (100% viability) and wells without cells as blank. Controls with DMSO and without drugs were also performed. Miltefosine was used as a standard drug. We selected the maximal tested concentration of 100 µg/mL for all antiparasitic assays and those compounds with IC_50_ values lower than 100 µg/mL were considered not active [33].

### 3.9. Determination of the Activity Against L. infantum-Intracellular Amastigotes

Peritoneal macrophages were obtained as described previously and *L. infantum* amastigotes were obtained from spleens of infected hamsters by differential centrifugation. Peritoneal macrophages were seeded at 1 × 10^5^ cells per well in Nunc™ 16-well slide chambers (Aldrich, St. Louis, MO, USA) for 24 h at 37 °C in a 5% CO_2_-humidified incubator. Amastigotes were added to macrophages at 10:1 ratio (amastigotes:macrophage) and incubated for 24 h. Non-internalized parasites were removed by washing twice with medium and the cells were then incubated with compounds in a range concentration of 100 to 0.78 μg/mL for 120 h at 37 °C in 5% CO_2_, using miltefosine as standard drug. At the end of the assay, the cells were fixed in methanol, stained with Giemsa and observed under a light microscope to determine the number of infected macrophages out of 400 cells [33].

### 3.10. Determination of the Activity Against Trypanosoma cruzi-Trypomastigotes

Compounds **1**–**3**, **1a**–**c**, **2a**–**d**, **3a**, and **3b** were dissolved in DMSO and diluted in RPMI-1640 medium to determine the 50% inhibitory concentration (IC_50_) as described above for the antileishmanial assay. Free trypomastigotes obtained from LLC-MK2 cultures were counted in a Neubauer hemocytometer and seeded at 1 × 10^6^/well in 96-well microplates. The tested compounds were incubated in a range concentration of 100 to 0.78 μg/mL for 24 h at 37 °C in a 5% CO_2_ humidified incubator, using benznidazole as standard drug. The viability of the trypomastigotes was verified by the MTT assay as described above [34,35].

### 3.11. Determination of the Cytotoxicity Against Mammalian Cells

The 50% cytotoxic concentration (CC_50_) was determined in NCTC clone 929 cells. NCTC cells were seeded at 6 × 10^4^ cells/well in 96-well microplates at 37 °C in a 5% CO_2_. The mammalian cells were incubated with tested compounds to the highest concentration of 200 μg/mL for 48 h at 37 °C, using miltefosine as standard drug. The viability of the cells was determined by MTT assay at 570 nm [36]. The selectivity index (SI) was determined considering the following equation: CC_50_ NCTC cells/IC_50_ parasites.

### 3.12. Statistical Analysis

The data obtained represent the mean and standard deviation of duplicate samples from at least three independent assays. The IC_50 _and CC_50 _values were calculated using sigmoid dose-response curves in Graph Pad Prism 5.0 software (GraphPad Software, San Diego, CA, USA).

### 3.13. Molecular Interaction Fields (MIF)

The structures were drawn using the Marvin Sketch v. 6.1.4 [37], and the three dimensional structures (3D) were generated using the software Standardizer v 6.1.4. [37]. The 3D structures were used as the initial structures to generate molecular descriptors employing the VolSurf+ v 1.0.7 program [27]. The descriptors were generated using the following probes N1 (amide nitrogen-hydrogen bond donor probe), O (carbonyl oxygen-hydrogen bond acceptor probe), OH2 (water probe), and DRY (hydrophobic probe) [27], and other non MIF-derived descriptors, totalizing 128 descriptors.

### 3.14. Decision Tree

Knime 2.7.1 software (KNIME 2.7.1 the Konstanz Information Miner Copyright, 2003–2013) [38] was used to perform all the analyses described hereafter. The descriptors and class variables were imported from Volsurf + v.1.0.7 program. For internal validation we used cross-validation by the leave-one-out method, using the decision tree learning J48 (C4.5 decision tree) [28]. The compounds without activity at 100 µg/mL were classified as inactive and the remaining as active. Decision trees arrange a subset of descriptor components in a hierarchical fashion (a binary tree) such that on a particular node in the tree, a classification on a single component decides whether the left or the right branch underneath is followed. Decision trees provide both rules, and the means to associate specific molecular features/descriptors. The following optimal parameters were used: confidence factor = 0.25; minimal number of objects = 2; number of folds = 3; and seed = 1.

## 4. Conclusions

The results presented herein indicate that natural tirucallane triterpenoids **1**–**3** could be used as new prototypes for drug design studies against leishmaniasis and Chagas’ disease. Additionally, some important relationships between chemical structure and biological activity of these compounds could be established since several derivatives (compounds **1a**–**c**, **2a**–**d**, **3a** and **3b**) were prepared and assayed. The obtained data suggest that the presence of carbonyl or hydroxyl at C-3, and double bond between C-24 and C-25 with carboxylic acid at C-27 are crucial to the antileishmanial activity, while the carbonyl group at C-3 combined with a double bond (*Z* configuration) between C-24 and C-25, or with an ester group at C-27, or without a double bond between C-24 and C-25 cause reduction in antitrypanosomal activity. Therefore, complementary studies aiming at the determination of mechanism of action of the most active compounds **1**, **2**, **2a** and **3** should be conducted.
